# Effect of Dexmedetomidine on Biochemical Recurrence in Patients after Robot-Assisted Laparoscopic Radical Prostatectomy: A Retrospective Study

**DOI:** 10.3390/jpm11090912

**Published:** 2021-09-13

**Authors:** Young Chul Yoo, Won Sik Jang, Ki Jun Kim, Jung Hwa Hong, Sunmo Yang, Na Young Kim

**Affiliations:** 1 Department of Anesthesiology and Pain Medicine, Anesthesia and Pain Research Institute, Yonsei University College of Medicine, 50-1 Yonsei-ro, Seodaemun-gu, Seoul 03722, Korea; seaoyster@yuhs.ac (Y.C.Y.); kkj6063@yuhs.ac (K.J.K.); sunmong@yuhs.ac (S.Y.); 2 Department of Urology and Urological Science Institute, Yonsei University College of Medicine, 50-1 Yonsei-ro, Seodaemun-gu, Seoul 03722, Korea; sindakjang@yuhs.ac; 3 Department of Policy Research Affairs, National Health Insurance Service Ilsan Hospital, 100 Ilsan-ro, Ilsandong-gu, Goyang-si 10444, Gyeonggi-do, Korea; jh_hong@nhimc.or.kr

**Keywords:** biochemical recurrence, dexmedetomidine, prostate cancer, radiographic progression

## Abstract

The usage of dexmedetomidine during cancer surgery in current clinical practice is debatable, largely owing to the differing reports of its efficacy based on cancer type. This study aimed to investigate the effects of dexmedetomidine on biochemical recurrence (BCR) and radiographic progression in patients with prostate cancer, who have undergone robot-assisted laparoscopic radical prostatectomy (RALP). Using follow-up data from two prospective randomized controlled studies, BCR and radiographic progression were compared between individuals who received dexmedetomidine (*n* = 58) and those who received saline (*n* = 56). Patients with complete follow-up records between July 2013 and June 2019 were enrolled in this study. There were no significant between-group differences in the number of patients who developed BCR and those who showed positive radiographic progression. Based on the Cox regression analysis, age (*p* = 0.015), Gleason score ≥ 8 (*p* < 0.001), and pathological tumor stage 3a and 3b (both *p* < 0.001) were shown to be significant predictors of post-RALP BCR. However, there was no impact on the dexmedetomidine or control groups. Low-dose administration of dexmedetomidine at a rate of 0.3–0.4 μg/kg/h did not significantly affect BCR incidence following RALP. In addition, no beneficial effect was noted on radiographic progression.

## 1. Introduction

Several reports have highlighted the importance of understanding and defining various factors during the perioperative period that may affect the postoperative long-term outcomes of patients with cancer [1,2]. Although the perioperative period is relatively short, it involves numerous risk factors and presents unexploited opportunities for improving the overall patient survival [1]. The anesthetic and analgesic approaches are prominent aspects of surgery that may be manipulated to improve various endocrinological, immunological, and cancer-related outcomes [2,3,4].

Recently, clinical studies on the effect of dexmedetomidine, an alpha-2 adrenoreceptor agonist commonly used in the perioperative period, have been performed for several types of cancer. The effects elicited by dexmedetomidine, particularly the sympatholytic effects, are suggestive of its benefits in the perioperative care of patients with cancer [5,6]. Further, a recent in vivo study has shown that dexmedetomidine inhibits esophageal cancer progression via miR-143-3P/epidermal growth factor receptor pathway substrate 8 [7]. However, results of in vitro and in vivo studies of dexmedetomidine revealed that tumor cell proliferation and metastasis are promoted, and overall survival is reduced [8,9,10,11,12,13,14,15,16,17,18]. In contrast, Owuso et al. reported that dexmedetomidine did not affect the survival of patients who underwent cytoreductive surgery with hyperthermic intraperitoneal chemotherapy for peritoneal carcinomatosis [19]. Hence, with variable results reported for different types of cancers, the use of dexmedetomidine during cancer surgery is currently debatable; this may be attributed to a lack of robust prospective data.

We previously conducted two prospective, double-blinded, randomized controlled trials to evaluate the effect of dexmedetomidine on intraocular pressure (IOP) and heart-rate corrected QT (QTc) intervals during robot-assisted laparoscopic radical prostatectomy (RALP) [20,21]. The patients included in the aforementioned studies had undergone RALP 4–6 years ago, which allowed us to assess their cancer status and long-term outcome. However, to the best of our knowledge, no study has investigated the effects of dexmedetomidine on prostate cancer recurrence after RALP. Therefore, this study aimed to investigate the effects of dexmedetomidine on biochemical recurrence (BCR), which reflects the long-term oncologic outcomes of prostate cancer in the patients who underwent RALP. Further, radiographic progression was also evaluated.

## 2. Materials and Methods

### 2.1. Study Design and Participants

The protocols of the double-blinded, randomized controlled IOP and QTc trials, which evaluated the effect of dexmedetomidine on IOP and QTc intervals in patients who had undergone RALP, have been described previously [20,21]. Sixty-seven patients were enrolled between July and December 2013 for the IOP trial, and 47 patients were enrolled between August 2015 and January 2016 for the QTc trial. We obtained additional ethics approvals from the Institutional Review Board (IRB) and Hospital Research Ethics Committee (Yonsei University Health System, Seoul, Korea; IRB protocol No. 4-2019-0424) for this follow-up study. This study was performed in accordance with relevant guidelines and regulations. The requirement for informed consent from the patients was waived. In this study, patients with complete follow-up records between July 2013 and June 2019 were enrolled. Patients who have been treated for other cancers or have incomplete follow-up data were excluded. For the current study, BCR, radiographic progression status, initial prostate-specific antigen (PSA) levels, Gleason score, tumor volume, surgical margin status, pathological tumor stage, and lymph node metastasis were included as variables in this study. Gleason score was assigned based on the 2005 International Society of Urological Pathology Modified Gleason System [22]. Pathological tumor stage and lymph node metastasis (TNM) was determined according to the 7th edition American Joint Committee on Cancer TNM staging system [23].

### 2.2. Anesthesia Protocol

Depending on the group, dexmedetomidine (*n* = 58) (100 mg/mL in a 2-mL vial; Hospira Worldwide, Seoul, Korea) or saline (*n* = 56) was administered at a rate of 0.3–0.4 μg/kg/hour from the initiation of anesthesia until the end of pneumoperitoneum maintenance. All patients underwent conventional inhalation anesthesia and were treated using the same modality. Anesthesia induction was performed using propofol (1.5–2 mg/kg) and rocuronium (0.6–1.2 mg/kg) and was maintained with sevoflurane (0.6–2.3 age-adjusted minimal alveolar concentration) and remifentanil (0.02–0.1 μg/kg/min). After induction, pneumoperitoneum was maintained at 15 mm Hg carbon dioxide in the steep (29°) Trendelenburg position.

### 2.3. Follow-up

For follow-up, the patients returned to the clinic at 3-month intervals for the first 2 years, and then at 6-month intervals for the next 3 years. Thereafter, annual follow-ups for PSA monitoring were recommended. BCR was defined as any two consecutive increases of ≥0.2 ng/mL in serum PSA levels following RALP [24]. If a patient showed symptomatic progression or an increase in PSA levels, follow-up imaging studies, including whole-body bone scan and abdominal-pelvic computed tomography or magnetic resonance imaging, were performed. Radiographic progression-free survival was defined as the duration from RALP to either disease progression based on the imaging studies, or to death as a result of any cause.

### 2.4. Statistical Analysis

Continuous and categorical variables were presented as mean (±standard deviation) and n (%), respectively. Between-group comparisons were performed using an independent two-sample *t*-test and a chi-square test, respectively. The log-rank test and Kaplan-Meier curve analysis were used for between-group comparisons of post-RALP BCR and radiographic progression-free survival for up to 60 months. Additionally, univariate and multivariate Cox regression analyses were performed to determine the factors that affected post-RALP BCR and radiographic progression. All statistical analyses were performed using Statistical Analysis Software version 9.4.

## 3. Results

**Figure 1:**
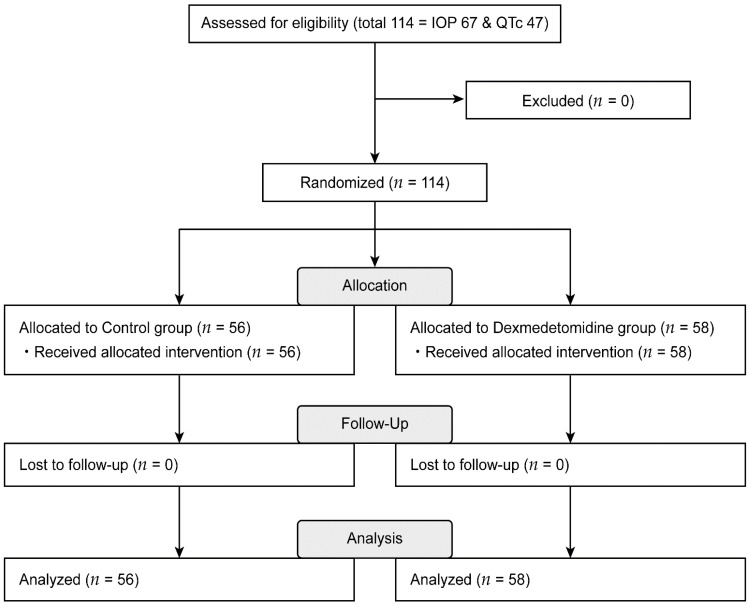
Consort flow diagram. IOP = intraocular pressure; QTc = heart-rate corrected QT interval.

Of the original 114 eligible patients, 67 and 47 patients were included in the IOP and QTc trials, respectively, with none of the patients being excluded due to loss to follow-up. Finally, 56 and 58 patients were allocated to the control and dexmedetomidine groups, respectively (Figure 1).

There were no significant between-group differences in the demographics and operative variables, except for the administered remifentanil dose (Table 1); the intraoperative administered dose of remifentanil was significantly lower in the dexmedetomidine group than in the control group (492 ± 169 vs. 731 ± 269 μg; *p* < 0.001).

Table 2 demonstrates the postoperative outcomes and pathologic variables. There were no significant between-group differences in the number of patients who developed BCR and those who demonstrated positive radiographic progression. Other variables, including initial PSA levels, Gleason score, tumor volume, surgical margin status, pathological tumor stage, and lymph node metastasis did not show differences between the two groups.

**Figure 2:**
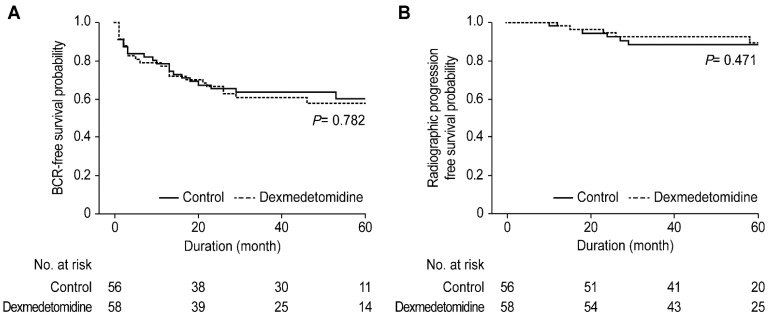
Biochemical recurrence-free survival (**A**) and radiographic progression-free survival (**B**) between the control and dexmedetomidine groups until the 60-month follow-up after robot-assisted laparoscopic radical prostatectomy.

The mean follow-up duration of BCR was 42 [(Interquartile range) IQR, 13–56)] months in the control group and 36 [IQR, 13–56] months in the dexmedetomidine group. In the radiographic progression, median follow-up duration in the control group was 52 [IQR, 35–65] and that in the dexmedetomidine group was 49 [IQR, 38–66] months. The BCR and radiographic progression-free survival for up to 60 months after RALP between the dexmedetomidine and control groups are demonstrated in Figure 2; no significant differences were observed between the two groups.

Other variables, including initial PSA levels, Gleason score, tumor volume, surgical margin status, pathological tumor stage, and lymph node metastasis did not show differences between the two groups.

The Cox regression analysis showed that age (Hazards ratio [HR] = 1.05, 95% confidence interval [CI]: 1.01–1.09; *p* = 0.015), Gleason score ≥ 8 (HR = 11.07, 95% CI: 3.81–32.19; *p* < 0.001), and pathological tumor stage 3a and 3b (HR = 4.92, 95% CI: 2.04–11.88, and HR = 8.86, 95% CI: 2.56–30.72, respectively; both *p* < 0.001) were significant predictors of post-RALP BCR; however, there was no effect on either the dexmedetomidine or control group (Table 3).

Further, the group was not an independent risk factor for radiographic progression after RALP; however, the following variables were significant predictors (Table 4): duration of postoperative hospital stay (HR = 1.43, 95% CI: 1.11–1.85; *p* = 0.007), and pathological tumor stage 3a and 3b (HR = 9.64, 95% CI: 1.38–67.25; *p* = 0.022, and HR = 17.61, 95% CI: 1.09–285.07, *p* = 0.044, respectively).

## 4. Discussion

This is the first randomized trial to demonstrate the effects of intraoperative continuous infusion of dexmedetomidine on long-term oncologic outcomes in patients with prostate cancer who have undergone RALP. Low-dose administration of dexmedetomidine at a rate of 0.3–0.4 μg/kg/h from the initiation of anesthesia to the end of pneumoperitoneum maintenance did not significantly affect the incidence of post-RALP BCR. Additionally, there was no beneficial effect on radiographic progression either.

The sympathetic and immunoregulatory effects of dexmedetomidine may underlie its perioperative benefits in patients with cancer. However, recent in vitro experiments have reported that dexmedetomidine may have unfavorable effects on patients with cancer, as observed in breast, lung, colon, and liver cancer cell lines [10,14,18]. The mechanisms underlying the effects of dexmedetomidine on tumor cells are as follows: it directly activates tumor alpha-2 adrenoreceptors [12,18], affects the capillary diameter or permeability of tumor cells [10], and drives microstructural changes in the tumor environment, causing proliferation, local invasion, and metastasis of tumor cells [11]. Additionally, current clinical evidence has raised concerns regarding the perioperative use of dexmedetomidine in patients with several types of cancer [14,15,16,17,18]. In breast cancer, dexmedetomidine has promoted cancer progression in vivo and in vitro by acting on alpha-2 adrenoreceptor and activating extracellular signal-related protein kinases [10,11,12,13,14]. Clinical study in lung cancer has shown that intraoperative administration of dexmedetomidine is associated with reduced overall survival rates [16]. Moreover, a study on a mouse model of lung cancer has demonstrated that dexmedetomidine promoted postoperative metastasis by augmenting the number of monocytic myeloid-derived suppressor cells (M-MDSCs) via alpha-2 adrenoreceptor stimulation [17], and in vitro study suggested that dexmedetomidine promotes cancer cell survival in lung cancer through signaling via on alpha-2 adrenoreceptor [18]. Further, a recent clinical study on hepatocellular carcinoma reported that dexmedetomidine did not significantly affect progression of malignancy; however, in case of activation of hepatic stellate cells, dexmedetomidine promoted tumor growth and metastasis [14].

It remains unclear whether dexmedetomidine affects the postoperative prognosis and outcomes in patients with all types of cancer. Recently, dexmedetomidine was reported to inhibit esophageal cancer progression via the miR-143-3P/epidermal growth factor receptor pathway substrate 8 in vivo [7]. Additionally, Owuso et al. performed a retrospective study on patients who had undergone cytoreductive surgery with hyperthermic intraperitoneal chemotherapy and demonstrated that intraoperative and/or early postoperative continuous infusion of dexmedetomidine was not associated with survival [19]. Apart from the present retrospective analysis and the aforementioned propensity score-matched retrospective study on patients with non-small cell lung cancer [16], there has not been any randomized trial to evaluate the influence of dexmedetomidine on long-term cancer outcomes.

Based on the data of the previous randomized controlled trials [19,20], this study showed that dexmedetomidine did not significantly affect BCR and radiographic progression in patients with prostate cancer within 4–6 years after RALP. Previous findings regarding this topic have been inconsistent, and the effect of dexmedetomidine on prostate cancer cell lines remains unclear. In breast, lung, and colon cancer [10,12,16], dexmedetomidine has been reported to be associated with pro-tumoral effects; however, it has shown to improve outcomes in patients with ovarian cancer by impeding with the activation of the chemotherapy drug-resistance pathway [25]. This suggests that dexmedetomidine may not exhibit similar pro-tumoral effects across all types of cancer. Therefore, the inconsistency between our findings and those of previous studies may be because this study is the first to examine the effects of dexmedetomidine on patients with prostate cancer. Furthermore, the average dose of total administered dexmedetomidine in this study was 56.4 μg (0.3–0.4 μg/kg/h; concentration of 4 μg/mL), which is lower than the dose administered in previous reports. Animal studies have shown that the metastasis-promoting effects of dexmedetomidine varied with the dose range. The range of 2.5–10 μg/kg/h has shown inconsistent results, while a higher dose range of 10–20 μg/kg/h has consistently shown deleterious effects [10]. The clinical reflection of the dose effects of dexmedetomidine observed in animal studies remains unclear; however, these findings suggest that the unfavorable effects of dexmedetomidine in patients may be dose-dependent. The median intraoperative administered doses of dexmedetomidine in the two previous trials on patients with lung cancer were 100 μg (57.47–140 μg) [16] and 122 μg (118–146 μg) [17], both of which are higher doses than that used in this study. Pascal et al., who reported findings that were consistent with our findings, used a dose of 0.1–0.7 μg/kg/h [19]. These inconsistent findings further indicate a mixed survival effect of low-dose dexmedetomidine.

This study has several limitations. First, generalization of our results may not be possible due to the selection bias commonly present in single-center retrospective studies. However, we may have limited the bias compared with other retrospective observational studies by controlling for other intraoperative variables in the study with the inclusion of randomly assigned patients. Second, this study had a limited sample size, given the original size of the IOP and QTc trials, which warrants the need for future prospective studies with a larger sample size.

## 5. Conclusions

In patients with prostate cancer who had undergone RALP, administration of low-dose dexmedetomidine at a rate of 0.3–0.4 μg/kg/h was not associated with a significant impact on BCR, which reflects the long-term oncologic outcomes of prostate cancer. Furthermore, dexmedetomidine did not affect the radiographic progression of prostate cancer following RALP. Further animal and prospective studies may be needed to further assess the effects of dexmedetomidine on prostate cancer.

## Figures and Tables

**Table 1:** Demographics and operative variables.

Variable	Control (*n* = 56)	Dexmedetomidine (*n* = 58)	*p* Value
Age (years)	64 ± 9	63 ± 8	0.686
Body mass index (kg/m^2^)	24.6 ± 2.1	23.9 ± 2.8	0.131
ASA physical status			0.412
I	19 (44%)	24 (56%)	
II	37 (52%)	34 (48%)	
Co-morbidities			0.500
Hypertension	24 (43%)	27 (47%)	
Diabetic mellitus	7 (13%)	5 (9%)	
Pneumoperitoneum duration (min)	117 ± 33	109 ± 34	0.202
Surgery duration (min)	150 ± 31	143 ± 34	0.246
Anesthesia duration (min)	206 ± 33	194 ± 34	0.075
Administered dose of remifentanil (μg)	731 ± 269	492 ± 169	<0.001 *
Administered dose of ephedrine (mg)	4 ± 7	5 ± 7	0.371
Intraoperative fluid input and output (mL)			
Crystalloid	1346 ± 345	1294 ± 597	0.566
Colloid	486 ± 184	522 ± 178	0.293
Blood loss	337 ± 179	271 ± 198	0.066
Urine output	352 ± 196	359 ± 224	0.867

Values are presented as mean ± standard deviation or number of patients (%). * *p* <0.05. ASA, American Society of Anesthesiologists.

**Table 2:** Postoperative outcomes and pathological variables.

Variable	Control (*n* = 56)	Dexmedetomidine (*n* = 58)	*p* Value
Biochemical recurrence	21 (38%)	23 (40%)	0.813
Positive radiographic progression	7 (13%)	5 (9%)	0.500
Postoperative hospital stays (days)	4 ± 2	4 ± 2	0.553
Initial PSA level (ng/mL)	13.5 ± 15.2	17.7 ± 21.0	0.218
Gleason score			0.038
6	28 (50%)	16 (28%)	
7	17 (30%)	29 (50%)	
≥8	11 (20%)	13 (22%)	
Tumor volume (cc)			0.976
<1	24 (43%)	24 (41%)	
≥1 and <5	23 (41%)	25 (43%)	
≥5	9 (16%)	9 (16%)	
Surgical margin status			0.394
Negative	39 (70%)	36 (62%)	
Positive	17 (30%)	22 (38%)	
Pathological tumor stage			0.967
2	37 (66%)	37 (64%)	
3a	11 (20%)	12 (21%)	
3b	8 (14%)	9 (16%)	
Lymph node metastasis			>0.999
Negative	54 (96%)	56 (97%)	
Positive	2 (4%)	2 (3%)	

Values are presented as mean ± standard deviation or number of patients (%). PSA, prostate specific antigen.

**Table 3:** Univariate and multivariate analyses of risk factors for biochemical recurrence after RALP (*n* = 114).

Variable	Univariate		Multivariate	
	HR (95% CI)	*p* Value	HR (95% CI)	*p* Value
Group				
Control	1		1	
Dexmedetomidine	1.09 (0.60–1.96)	0.786	0.80 (0.43–1.47)	0.468
Age (years)	1.04 (1.00–1.08)	0.038 *	1.05 (1.01–1.09)	0.015 *
Body mass index, kg/m^2^	1.09 (0.95–1.24)	0.227		
ASA physical status				
I	1			
II	1.82 (0.94–3.53)	0.078		
Co-morbidities				
Hypertension	1.32 (0.73–2.39)	0.353		
Diabetic mellitus	1.60 (0.67–3.78)	0.288		
Pneumoperitoneum duration (min)	0.99 (0.98–1.00)	0.099		
Surgery duration (min)	0.99 (0.98–1.00)	0.142		
Anesthesia duration (min)	0.99 (0.99–1.00)	0.259		
Administered dose of remifentanil (μg)	0.98 (0.96–1.01)	0.200		
Administered dose of ephedrine (mg)	1.03 (0.98–1.07)	0.162		
Intraoperative fluid input and output (mL)				
Crystalloid	1.00 (0.99–1.00)	0.444		
Colloid	1.00 (0.99–1.00)	0.816		
Blood loss	1.00 (1.00–1.01)	0.087		
Urine output	1.00 (0.99–1.00)	0.744		
Duration of postoperative hospital stay (days)	1.02 (0.88–1.18)	0.769		
Initial PSA level, ng/mL	1.03 (1.02–1.04)	<0.001 *	1.02 (0.99–1.03)	0.075
Gleason score				
6	1		1	
7	3.02 (1.18–7.73)	0.021 *	1.62 (0.59–4.46)	0.352
≥8	19.53 (7.74–49.31)	<0.001 *	11.07 (3.81–32.19)	<0.001 *
Tumor volume (cc)				
<1	1		1	
≥1 and <5	1.37 (0.65–2.88)	0.408	1.03 (0.45–2.40)	0.940
≥5	10.67 (4.83–23.58)	<0.001 *	1.36 (0.32–5.75)	0.674
Surgical margin status				
Negative	1		1	
Positive	4.38 (2.38–8.06)	<0.001 *	1.26 (0.53–3.01)	0.608
Pathological tumor stage				
2	1		1	
3a	5.72 (2.71–12.07)	<0.001 *	4.92 (2.04–11.88)	<0.001 *
3b	15.67 (7.26–33.82)	<0.001 *	8.86 (2.56–30.72)	<0.001 *
Lymph node metastasis				
Negative	1		1	
Positive	3.67 (1.13–11.95)	0.031 *	1.07 (0.24–4.78)	0.931

* *p* < 0.05. HR, hazards ratio; CI, confidence interval; ASA, American Society of Anesthesiologists; PSA, prostate specific antigen; RALP, robot-assisted laparoscopic radical prostatectomy.

**Table 4:** Univariate and multivariate analyses of risk factors for radiographic progression after RALP (*n* = 114).

Variable	Univariate		Multivariate	
	HR (95% CI)	*p* Value	HR (95% CI)	*p* Value
Group				
control	1		1	
dexmedetomidine	0.66 (0.21–2.07)	0.474	0.71 (0.21–2.39)	0.580
Age (years)	1.04 (0.97–1.11)	0.279		
Body mass index (kg/m^2^)	1.09 (0.87–1.36)	0.460		
ASA physical status				
I	1			
II	1.30 (0.39–4.30)	0.673		
Co-morbidities				
Hypertension	1.87 (0.59–5.91)	0.285		
Diabetic mellitus	2.05 (0.45–9.40)	0.355		
Pneumoperitoneum duration (min)	0.99 (0.97–1.01)	0.451		
Surgery duration (min)	1.00 (0.98–1.02)	0.937		
Anesthesia duration (min)	1.00 (0.99–1.02)	0.746		
Administered dose of remifentanil (μg)	1.01 (0.98–1.05)	0.505		
Administered dose of study drug (cc)	1.01 (0.86–1.18)	0.934		
Administered dose of ephedrine (mg)	1.03 (0.97–1.10)	0.334		
Intraoperative fluid input and output (mL)				
Crystalloid	1.00 (0.99 –1.00)	0.716		
Colloid	1.00 (0.99–1.00)	0.229		
Blood loss	1.00 (1.00–1.01)	0.063		
Urine output	1.00 (1.00–1.01)	0.962		
Postoperative hospital stays (days)	1.33 (1.08–1.65)	0.008 *	1.43 (1.11–1.85)	0.007 *
Initial PSA level (ng/mL)	1.02 (1.00–1.04)	0.034 *	1.01 (0.97–1.05)	0.632
Gleason score				
6	1		1	
7	6.95 (0.30–161.01)	0.227	2.74 (0.10–75.96)	0.553
8 or more	37.99 (1.86–776.45)	0.018 *	12.84 (0.54–305.92)	0.115
Tumor volume (cc)				
<1	1		1	
≥1 and less than 5	7.41 (0.89–61.71)	0.064	2.83 (0.42–19.35)	0.288
5 or more	18.24 (2.12–156.79)	0.008 *	0.23 (0.01–5.77)	0.373
Surgical margin status				
Negative	1			
Positive	2.18 (0.70–6.77)	0.179		
Pathological tumor stage				
2	1		1	
3a	16.54 (1.93–141.87)	0.011 *	9.64 (1.38–67.25)	0.022 *
3b	27.40 (3.30–227.71)	0.002 *	17.61 (1.09–285.07)	0.044 *
Lymph node metastasis				
Negative	1		1	
Positive	6.32 (1.37–29.06)	0.018 *	2.20 (0.23–20.85)	0.491

* *p* < 0.05. HR, hazards ratio; CI, confidence interval; ASA, American Society of Anesthesiologists; PSA, prostate specific antigen; RALP, robot-assisted laparoscopic radical prostatectomy.

## Data Availability

The datasets used and/or analyzed during the current study are available from the corresponding author on reasonable request.
